# Sepsis: New perspectives

**DOI:** 10.4103/2321-3868.137602

**Published:** 2014-07-28

**Authors:** David P. Mackie

**Affiliations:** Red Cross Hospital, Beverwijk, The Netherlands

This issue of *Burns & Trauma* is largely devoted to sepsis. The subject is of great importance, as is emphasized by the fact that much of our daily clinical work is aimed at preventing this potentially lethal condition. All hospital clinicians, and intensive care specialists in particular recognize the syndrome, but what is it, exactly, and how is it diagnosed? It is interesting that none of the excellent articles in this issue have attempted a definition, although prompt recognition and treatment are essential. Gray areas of Medicine, such as this, suggests that our understanding of the phenomenon is incomplete. For this reason, the wide-ranging, erudite articles contained in this journal are a welcome contribution to our current knowledge of the condition.

The article entitled “Prediction of sepsis in trauma patients” by He Jin and colleagues has direct clinical relevance. In this article, the authors have reviewed the biomarkers, clinical characteristics and demographic features associated with the condition. As they have pointed out, while blood cultures are considered the definitive diagnostic marker, results are subject to a time lag and a considerable proportion of patients with clinical sepsis, fail to provide positive blood culture results. Whether these patients are suffering from an invasion of micro-organisms, or whether a sepsis-like state can be induced some other disruption of the immune response remains an open question. However, few would argue with their conclusion that “the prediction of sepsis in trauma patients is still a challenge. Though approximately 180 biomarkers for sepsis have been reported, the studies performed on the biomarkers for post-traumatic sepsis are few and the results are controversial. Trauma can affect immunologic function, and injury characteristics, such as injury severity and the number of injuries are risk factors that are associated with sepsis following trauma.” These words reflect the assertion that the tools at our disposal for predicting sepsis are far from ideal.[1]

**Table 1:** 

Access this article online	
**Quick Response Code**:	**Website**: www.burnstrauma.com
	**DOI**: 10.4103/2321-3868.137602

In addition, there are 3 articles highlighting advances in different areas of the immune response to sepsis. The paper detailing advances in sepsis-associated liver dysfunction by Dawei Wang *et al.* is an excellent review of the effects of sepsis on liver function; a subject which has been largely ignored by clinicians, possibly because there is no definitive treatment for liver dysfunction. Nevertheless, the implications for detoxification processes are clear, especially for those drugs eliminated by hepatic metabolism. Perhaps more importantly, the article provides further insight into the destructive effects of sepsis on all major organ systems. This paper is recommended reading for clinicians and researchers alike.[2]

In a similar vein, the review of the characteristics of bone marrow tyrosine kinase, bone marrow kinase X-linked (BMX), by Le Qiu and colleagues from Anhui Province, provides a unique into the role of this enzyme in inflammatory disorders. This paper is especially welcome because much of the work described in the article has originated in the fields of oncology and chronic inflammatory states. The presentation of knowledge from different fields of medicine is frequently lacking in specialized research; while the clinical implications for the treatment of sepsis are not yet apparent, awareness of developments in this area may well trigger new approaches in time.[3]

The third review paper concerns the key role of plateletneutrophil interactions in sepsis. This article, by Xu Wang *et al.* is a timely review of the current state of knowledge of the essential role of platelets and neutrophils in the inflammatory response. While the importance of these cells in sepsis has been recognized for decades, insight into their actions and interactions has expanded significantly, in recent years. These advances are described eloquently and the implications for potential therapeutic interventions are clearly set out in this important review.[4]

The final paper in this issue concerning sepsis comprises a prospective study into the clinical effects of continuous veno-veno hemofiltration (CVVH) in burn patients with sepsis. This study is a remarkable achievement and Cheng Xu and his colleagues are to be congratulated in carrying out this trial. The study concentrated on the effects of CVVH on plasma levels of interleukin (IL)-6, IL-8 and tumor necrosis factor (TNF)-α, which were significantly reduced in the dialyzed group, but clinical effects were also apparent, although mortality was low in both groups. This investigation is an important contribution to the clinical dilemma if and when patients should be exposed to the major intervention of CVVH, when sepsis supervenes. Until now, most physicians would wait until frank signs of renal dysfunction become manifest. This paper forms a strong argument for earlier application of hemofiltration.[5]

Together, the articles included in this issue comprise a major contribution to our understanding of sepsis, as well as indicating potential and actual therapeutic responses. I congratulate all the authors on their outstanding efforts, as well as the guest editor Prof. Yongming Yao for collecting these papers together. The issue deserves to be read widely.

## Announcement

### Ipad App

A free application to browse and search the journal’s content is now available for iPad. The application provides “Table of Contents” of the last issues, which are stored on the device for future offline browsing. Internet connection is required to access the back issues and search facility. The application is compatible with iPad and requires iOS 3.1 or later. The application can be downloaded from https://itunes.apple.com/au/app/burns-trauma/id884983755?mt=8&ign-mpt=uo=2. Scan the QR code to download the *Burns & Trauma* iPad app instantly! For suggestions and comments do write back to us.
